# App‐based mindfulness meditation reduces perceived stress and improves self‐regulation in working university students: A randomised controlled trial

**DOI:** 10.1111/aphw.12328

**Published:** 2021-12-27

**Authors:** Poul Maria Schulte‐Frankenfeld, Fynn‐Mathis Trautwein

**Affiliations:** ^1^ Department of Psychology University of Amsterdam Amsterdam The Netherlands; ^2^ Department of Psychosomatic Medicine and Psychotherapy, Medical Center, Faculty of Medicine University of Freiburg Freiburg im Breisgau Germany; ^3^ Institute for Frontier Areas of Psychology and Mental Health Freiburg im Breisgau Germany

**Keywords:** cognitive reappraisal, digital treatments, meditation, mindfulness, self‐regulation, stress

## Abstract

We investigated whether a brief mindfulness meditation programme delivered via a smartphone app improves perceived stress, self‐regulation and life satisfaction in part‐time working university students. Mindfulness and cognitive reappraisal were analysed as potential mediators. A total of 64 university students working at least 20 h per week during the semester were randomised to either a mindfulness‐based mobile intervention or a wait‐list control condition. Participants in the intervention group were asked to complete one training session of 10–15 min per day using the app. Psychological measures were assessed via a self‐report questionnaire at baseline and after 8 weeks. The online mindfulness programme was found to significantly decrease perceived stress (*η*_*p*_^*2*^ = .180, a large effect). It also increased self‐regulation (*η*_*p*_^*2*^ = .195, a large effect), mindfulness (*η*_*p*_^*2*^ = .174; a large effect) and cognitive reappraisal (*η*_*p*_^*2*^ = .136, a medium effect). Increments in life satisfaction were not significant. Changes in self‐regulation were mediated by increased mindfulness. Overall, the mobile application was effective in improving mental well‐being and coping abilities in a non‐clinical sample, encouraging further development of digital health treatments.

## INTRODUCTION

Meditation and mindfulness‐based interventions are well‐established treatments for increasing quality of life and well‐being in non‐clinical samples (Blanck et al., 2018; Eberth & Sedlmeier, 2012; Khoury et al., 2015) and for treating a wide range of mental conditions (Goyal et al., 2014; Grossman et al., 2004). They have been shown to reduce symptoms of anxiety, depression and stress (González‐Valero et al., 2019; Lomas et al., 2019; Querstret et al., 2020), improve body awareness (Treves et al., 2019) and regulation (Bornemann et al., 2019; Kadziolka et al., 2016) and foster physical health (Black & Slavich, 2016; Creswell et al., 2019). In recent years, mobile applications have become a possible form of delivery. Most smartphone‐based solutions currently available offer well‐structured programmes, accessible on demand and from everywhere in the world. Given their flexibility and autonomy, digital courses appear to be particularly valuable for individuals living busy and active lives, in remote areas and for times when in‐person meetings are not available.

Although mobile mindfulness applications are gaining in popularity, we have only limited knowledge about their efficacy. Some studies found small to medium effects for reducing symptoms of depression, anxiety and stress (Bostock et al., 2019; Fish & Saul, 2019; Flett et al., 2019; Huberty et al., 2019), and early meta‐analyses seem to support these findings (Firth et al., 2017; Mani et al., 2015; Spijkerman et al., 2016). However, the overall body of literature focusing on the efficacy of smartphone‐based mindfulness training is still small and limited in regard to investigated populations and outcome measures (van Emmerik et al., 2020).

We thus used a randomised wait‐list controlled trial with pre–post measurement to focus on a population previously ignored by mindfulness research, yet particularly prone to psychological strain — part‐time working university students. Several studies suggest that university students working 20 or more hours per week show inferior academic performance (Curtis & Shani, 2002; Darolia, 2014; Krings et al., 2018; Triventi, 2014) as well as poorer physical health and lower mental well‐being (Broadbridge & Swanson, 2005; Carney et al., 2005; Park & Sprung, 2013). Previous studies among other highly demanded populations suggest that mindfulness‐based treatments can be effective in reducing distress and increasing well‐being (Ghawadra et al., 2019; Yang et al., 2018) as well as cognitive performance (Coo & Salanova, 2018; Zanesco et al., 2019). Thus, engaging in a brief mindfulness programme could be particularly beneficial for working university students.

Mindfulness has been defined as a mode of awareness evoked when attention is directed purposefully to the immediate experience while perpetuating a mental stance of acceptance (Bishop et al., 2004). Whereas mindfulness is assumed to be a common capacity of humans, the ability to deliberately evoke a mode of mindfulness in situations in which it would be beneficial can be improved through training. Mechanisms proposed to underlie this ability include enhanced awareness of present‐moment experiences (‘attention monitoring’) and an affective processing style oriented towards acceptance (‘acceptance’) (Lindsay & Creswell, 2017). When strengthened through mindfulness training, these two mechanisms are theorised to result in reduced stress and improved mental well‐being, improved cognitive functioning, improved emotion regulation and increased overall satisfaction with life (Lindsay & Creswell, 2017, 2019).

Although these findings indicate a good condition‐treatment match, the current trial is, to our best knowledge, the first to specifically examine potential effects of a mindfulness‐based intervention on working students. We were thus primarily interested in the effects of this intervention on perceived stress as a global indicator of mental well‐being (Kötter et al., 2019). Results from previous studies in clinical and healthy populations suggest that mindfulness‐based interventions are effective in reducing perceived stress (Eberth & Sedlmeier, 2012; Khoury et al., 2015), possibly driven by increases in acceptance orientation (Lindsay et al., 2018). We were further interested in self‐regulation as a central characteristic of cognitive functioning that is highly relevant for the working students' lives (Malanchini et al., 2019). Improved attention monitoring and self‐regulation of attention could help students to successfully manage a higher amount of demanding tasks by increasing task persistence (Valenzuela et al., 2020) and engaging in rather problem‐focused than emotion‐focused coping (La Fuente et al., 2018; Leyland et al., 2019), which could indirectly improve academic achievements and mental health (Pandey et al., 2018; Sahranavard et al., 2018). In other populations, mindfulness‐based interventions have shown to be effective in increasing self‐regulation (Rupprecht et al., 2017; Slutsky et al., 2019). Lastly, we were interested in the effects of the mindfulness‐based intervention on students' general life satisfaction. A mindfulness intervention may influence life satisfaction through heightened monitoring of positive life events (Lindsay & Creswell, 2017) and reappraisal of negative autobiographical memories (Gupta & Verma, 2020) and indirectly through its stress‐buffering effect (Bergin & Pakenham, 2016). Thus, we expected to see positive effects of the mobile mindfulness‐based intervention in a part‐time working students' population compared with a control condition on the following variables, defined as the primary outcomes: perceived stress, self‐regulation and life satisfaction.

We were further interested in examining how the mobile mindfulness‐based intervention yields its effects. To investigate whether changes in our primary outcome variables were driven by increased mindfulness, we used a common standard measure of mindfulness (Walach et al., 2006). We expected to see increased post‐intervention levels of mindfulness in the intervention condition compared with the control condition, and we expected changes in the primary outcomes to be meditated by changes in mindfulness. Additionally, we were interested in changes in the utilisation of particular emotion regulation strategies, as recent research suggests that improved emotion regulation might mediate the positive effects of mindfulness‐based interventions on mental well‐being in meditation novices (Freudenthaler et al., 2017; Garland et al., 2011; Hanley et al., 2015; Hanley & Garland, 2014). Although the primary focus of mindfulness‐based interventions is to develop a mental stance of acceptance and non‐interference, the involved cognitive and neural processes are thought to partially overlap with other emotion regulation strategies such as cognitive reappraisal (Dahl et al., 2015; Hölzel et al., 2011), as supported by empirical evidence (Ives‐Deliperi et al., 2011; Opialla et al., 2015). Thus, in addition to mindfulness, we investigated the role of cognitive reappraisal strategies as potential mediators to better characterise underlying mechanisms of the intervention effects and expected to see an increase in the utilisation of reappraisal strategies in the intervention condition compared with the control condition that would partly mediate the primary outcomes. Finally, we sought to analyse if potential improvements in the primary outcomes would depend on the amount of completed meditation sessions, as recent research reports mixed results for the dose–response relationship of mindfulness‐based programmes (Strohmaier, 2020), suggesting that very brief programmes might already be effective (Jones et al., 2021).

To test the hypotheses described above, we conducted a randomised wait‐list controlled trial with pre–post measurements over 8 weeks. Participants in the intervention condition were asked to engage in brief daily meditation sessions using a training plan assembled and delivered through the popular meditation app Balloon (G+J Innovation GmbH, 2019), which provides scientifically based secular meditation and mindfulness practices to a German‐speaking audience. A complete intervention plan specifying components of the training is available in the Supporting Information to this article.

## METHODS

### Participants

A convenience sample was recruited by disseminating invitations to participate in the form of flyers, posters, mailings and posts on social media primarily among the academic community, with a focus on channels and institutions offering programmes and services to working students, such as special LinkedIn groups or the FOM University of Applied Science. An online tool was open for registration from the 3rd till the 13th of October 2019. This registration period was pre‐determined due to the strict timeline of this trial, and the number of included participants was only restricted by this time limit. A total of 166 potential participants filled out the online recruitment questionnaire. Eligible test persons were (a) German‐speaking (b) students currently enrolled at a higher education institution and at the same time (c) employed at a company or institution with a minimum of 20 working hours per week, who (d) had not engaged in any mindfulness or relaxation practice on a daily basis during the past 6 months. Further, subjects who did not own (e) an Android or iOS device, (f) were younger than 18 years or (g) did not sign the informed consent were excluded.

**FIGURE 1:**
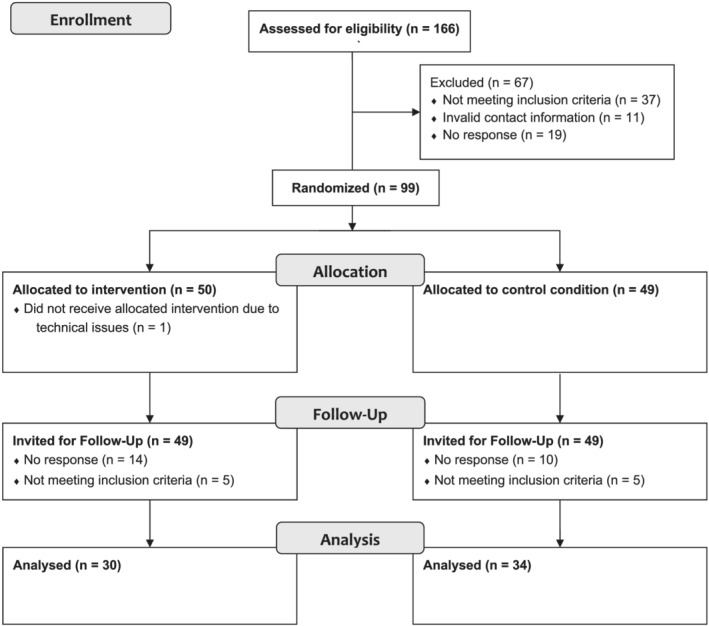
Selection of sample included in the final analysis

We screened all 166 candidates, of which 118 were found to be eligible. Participants who did not complete the baseline questionnaire (*n* = 19) were excluded and the remaining subsequently randomised to either the intervention group (*n* = 50) or the control group (*n* = 49). To ensure assessment of effects in the targeted population of part‐time working students, we further excluded participants who indicated a weekly workload of less than 20 hours despite asserting differently in the screening questionnaire (intervention group, *n* = 5; control group, *n* = 5). Furthermore, participants who had technical issues using the app (intervention group, *n* = 1) and who did not complete the post‐intervention questionnaire (intervention group, *n* = 14; control group, *n* = 10) were excluded. A total of 64 participants were included in the analysis (intervention group, *N* = 30; control group, *N* = 34; see Figure 1). Indicative sensitivity analysis performed with G*power shows that this convenience sample would be sufficiently strong to detect effects of *f*(V) = .398 with Pillais V = .137 in a multivariate repeated‐measures ANOVA with power of (1 − β) = 80%, and meta‐analyses reporting common effect sizes of hedge's *g* around .4 give reason to expect such medium effects (Khoury et al., 2015; Spijkerman et al., 2016).

To ensure blinded randomisation, eligible participants contact information was uploaded to a protected research environment at SoSci Survey (soscisurvey.de) and assigned randomly generated IDs, which were then paired with an independently randomised sequence of group assignments generated using randomisation software (randomizer.com) with a 1:1 group allocation ratio. To ensure data protection and prevent bias, only pseudonymised data were analysed and kept separately from clear data and contact information.

### Intervention and procedure

After filling out the Internet‐based baseline questionnaire, participants randomised to the intervention group immediately received an email with instructions to set up the Balloon app and complete a predefined 8‐week plan of six courses, a total of 57 sessions of each approximately 10 min. They were free to take more courses than those planned if wished. Participants could choose to take the daily session at any time or place during each day. When daily meditation was missed out, participants were encouraged to compensate for this as soon as possible with a double session.

The training course particularly assembled for this study was designed to cover major principles of mindfulness, stress and coping, happiness in live, meta‐cognition, self‐care and compassion. Each daily session covered essential aspects of mindfulness and meditation such as using breathing and other body sensation to ground awareness in the present moment, observing thoughts and feelings and cultivating benevolence and care towards oneself and others (see Supporting Information to this article for the detailed training plan). At the last day of intervention, participants of both groups received an invitation with a personalised link to fill out the Internet‐based post‐intervention questionnaire. Participants in the wait‐list control group were asked not to engage in any new mindfulness or relaxation activities during the intervention period. After completing the post‐intervention questionnaire, control group participants received access to the app.

### Measures and statistical analysis

Both the intervention and the wait‐list group were administered two online questionnaires, one at baseline and one at post‐intervention after 8 weeks, to collect data on perceived stress, self‐regulation and life satisfaction as primary outcome variables and mindfulness and cognitive reappraisal as secondary outcome variables. A scale measuring socially desired response tendencies was integrated to control for demand effects. The amount of successfully completed meditation sessions per participant was extracted from the backend database of the Balloon app. The analysed data set is archived in the Open Science Framework repository and openly available (https://doi.org/10.17605/OSF.IO/WUJSG).

#### Perceived stress

Perceived stress was assessed using the German version of the Perceived Stress Scale (PSS‐10) (Cohen, 2017). The 10‐item inventory measures the degree to which certain everyday occasions, such as unexpected occurrences, are being appraised as stressful. Responses are given on a 5‐point Likert scale ranging from 0 (*never*, *ger.: nie*) to 4 (*very often*, *ger.: sehr oft*). Scores are calculated as a sum of all items, ranging from 0 to 40 with higher scores indicating higher levels of perceived stress. The PSS has shown to be reliable in a US community sample and has been used in various studies in the field of stress research (Cohen, 1988). The German adaption has shown to be equally reliable and appropriate among a German community sample (Klein et al., 2016). In the present study, Cronbach's alpha for baseline PSS‐10 scores was .831.

#### Self‐regulation

Self‐regulation ability was assessed using the self‐regulation scale (SRS‐10) (Schwarzer, 2000). The 10‐item inventory measures the degree to which participants agree with situational statements about their own volition control capacity. Responses are assessed on a 4‐point Likert scale ranging from 1 (*not at all true*, *ger.: stimmt nicht*) to 4 (*exactly true*, *ger.: stimmt genau*). Scores are calculated as a sum of all items, ranging from 10 to 40 with higher totals indicating higher levels of self‐regulation ability. In the present study, Cronbach's alpha for baseline SRS‐10 scores was .784.

#### Life satisfaction

To assess general life satisfaction and happiness, the Questionnaire for the Assessment of Happiness (*ger.: Lebensglückskala*, LSS) was used (Ciccarello & Reinhard, 2014). The 9‐item inventory measures affective and cognitive components of happiness and life satisfaction from a bitemporal perspective, represented by a subscale for past life satisfaction (LSS‐past) consisting of six items and a subscale for current life satisfaction (LSS‐current) consisting of three items. Further, total life satisfaction (LSS‐total) can be reported as a sum of both subscales. Responses are recorded on a 9‐point Likert scale ranging from 1 (*negative appraisal*) to 9 (*positive appraisal*) with varying scale labels, such as *very unhappy* to *very happy* or *seldomly* to *very often*. Scores are the sums of all items in each subscale, ranging from 6 to 54 for past life satisfaction and 3 to 27 for current life satisfaction, or 9 to 81 for total life satisfaction, divided by the total amount of items. In the present study, Cronbach's alpha for baseline LSS‐total was found to be .926, for LSS‐past .918 and for LSS‐current .906.

#### Mindfulness

As a secondary outcome and mediation variable, levels of mindfulness were assessed using the German version of the Freiburg Mindfulness Inventory (FMI‐14) (Buchheld & Walach, 2002), ascertaining components of mindfulness understood as a holistic secular concept. The 14‐item inventory measures degrees to which subjects agree to statements about their experience of mindfulness states and traits in everyday life, covering major aspects of mindfulness such as presence and acceptance, on a 4‐point Likert scale ranging from 1 (*rarely*, *ger.: fast nie*) to 4 (*almost always*, *ger.: fast immer*). Thus, scores can range from 14 to 56 with higher scores indicating higher levels of mindfulness. Among general population and clinical samples, the FMI‐14 has shown to be valid and reliable (Walach et al., 2006). In the present study, Cronbach's alpha for baseline FMI‐14 scores was .834.

#### Emotion regulation

Preferences for the utilisation of different emotion regulation strategies was assessed using the German version of the Emotion Regulation Questionnaire (ERQ) (Abler & Kessler, 2011) based on the English questionnaire by Gross and John (2003). The 10‐item inventory measures the self‐assessed preference for either reappraising or suppressing regulation strategies as reactional patterns to emotional stimuli. Responses are assessed on a 7‐point Likert scale ranging from 1 (*strongly disagree*, *ger.: stimmt überhaupt nicht*) to 7 (*strongly agree*, *ger.: stimmt vollkommen*), with scores being calculated as sums of items in two subscales. The subscale for reappraisal consists of six items such as ‘If I want to experience more positive feelings, I change what I think of’, ranging from a total score of 6 to 42, whereas the subscale for suppression consists of four items such as ‘I am keeping my emotions to myself’ ranging from a total score of 4 to 28. In the present study, Cronbach's alpha for baseline EQR‐reappraisal was found to be .761 and for EQR‐suppression .640.

#### Social desirability

A short‐form scale to detect test forgery through positive self‐representation and socially desirable response tendencies (SEA) (Satow, 2012) was included at baseline. The 2‐item inventory measures degrees to which subjects agree to statements that have been found to be highly correlated with a tendency of ‘faking good’ in a calibration study on a 4‐point Likert scale ranging from 1 (*strongly disagree*, *ger.: trifft gar nicht zu*) to 4 (*strongly agree*, *ger.: trifft genau zu*). Thus, scores can range from 2 to 8 with higher scores indicating higher levels of socially desirable response tendencies. In a validation study, individual scores of 7 or higher were found to be a critical norm of faking good, although interpretations should consider general sample scores and be made on a case‐by‐case basis (Satow, 2012). In the present study, Cronbach's alpha for SEA scores was .697.

#### Sessions

The amount of meditation sessions per participant was extracted from the backend database of the Balloon app. A course element needed to be clicked on and listened to for several seconds before it was counted as a session by the system.

#### Statistical analysis

Overall intervention effects on perceived stress, self‐regulation and life satisfaction as primary outcome variables were assessed using a multivariate analysis of variances (MANOVA) with Pillai's trace as the test statistic, in which time (baseline vs. post‐intervention) was included as a within‐subject factor and group (intervention group vs. control group) was included as a between‐subject factor. Univariate tests were performed post hoc to assess group‐by‐time interaction effects on singular variables. Additional univariate tests were performed for mindfulness and emotion regulation as secondary outcome variables.

Furthermore, we tested mediating effects of mindfulness and emotion regulation on significant primary outcome variables by using a two‐condition within‐participant statistical mediation pathway analysis estimating the indirect effect of an independent on a dependent variable through a mediator, as described in Montoya and Hayes (2017). This statistical method differs in so far from commonly applied earlier approaches to within‐participant mediation analyses (Judd et al., 2001), as it does not rely on serial significance tests for discrete components of the mediation process, but rather compares the full indirect effect of one or more mediation paths to the direct and total effect of the independent on the dependent variable. Here, the indirect effect of each mediation path is estimated from the product of its sub‐paths, eliminating the need for multiple hypothesis testing. Finally, to test for potentially moderating dose–response effects of the number of completed mindfulness sessions, we ran separate regression models in the intervention group for each of the three outcome measures using a within‐participant statistical analysis, which estimated the interaction between sessions and change in the outcome variable over time (Montoya, 2019).

Statistical significance was set to α < .05 for all tests performed. Partial eta squared (η_p_^2^) is reported as the effect size indicator. All statistical calculations were performed using SPSS Version 27.0.0.0. An RStudio script developed by Allen et al. (2019) was used to create raincloud plots for data visualisation. Mediation and moderation calculations were performed using the MEMORE macro for SPSS in Version 2.1 (Montoya & Hayes, 2017; Montoya, 2019).

## RESULTS

### Sample description

At baseline, participants' mean age was 24.75 years (*SD* = 5.42, *range*: 18–48 years), and 64.1% were female. With 87.5%, the majority (*n* = 56) was currently enrolled in a bachelor's degree programme, few (*n* = 7) were pursuing a master's degree, and one participant did not indicate enrolment status. In sum, participants had a mean working load of 34.47 hours per week, (*SD* = 8.33, *range*: 20–50 h), and the majority (62.5%) had no previous experience with any mindfulness or relaxation techniques.

Detailed group characteristics are presented in Table 1. Prior to the assessment of intervention effects, quality of responses was controlled using SKA‐2 results. Few cases (*n* = 2) with a moderately critical score of 7 were found. As the mean score of 3.72 (*SD* = 1.36, *range*: 2–7) indicates a good overall response quality and critical scores of 8 were not reached, exclusion of these two cases was rejected.

**TABLE 1:** Demographic characteristics of study participants

	Randomised (*n* = 99)		Analysed (*n* = 64)	
Variables	Intervention group (*n* = 50)	Control group (*n* = 49)	Intervention group (*n* = 30)	Control group (*n* = 34)
Age, in years, *M* (*SD*)	25 (6.9)	24.6 (5.2)	24.5 (5.9)	25 (5.1)
Sex, female, *n* (%)	34 (69.4%)	33 (66%)	19 (63.3%)	22 (64.7%)
Education, bachelor's programme, *n* (%)	46 (93.9%)	42 (84%)	28 (93.3%)	28 (82.4%)
Work per week, in hours, *M* (*SD*)	30.6 (12.2)	32.3 (10.8)	33.8 (8.6)	35 (8.1)
Previous experience with mindfulness or relaxation techniques, yes, *n* (%)	18 (36.7%)	23 (46%)	11 (36.7%)	13 (38.2%)

### Intervention effects

**FIGURE 2:**
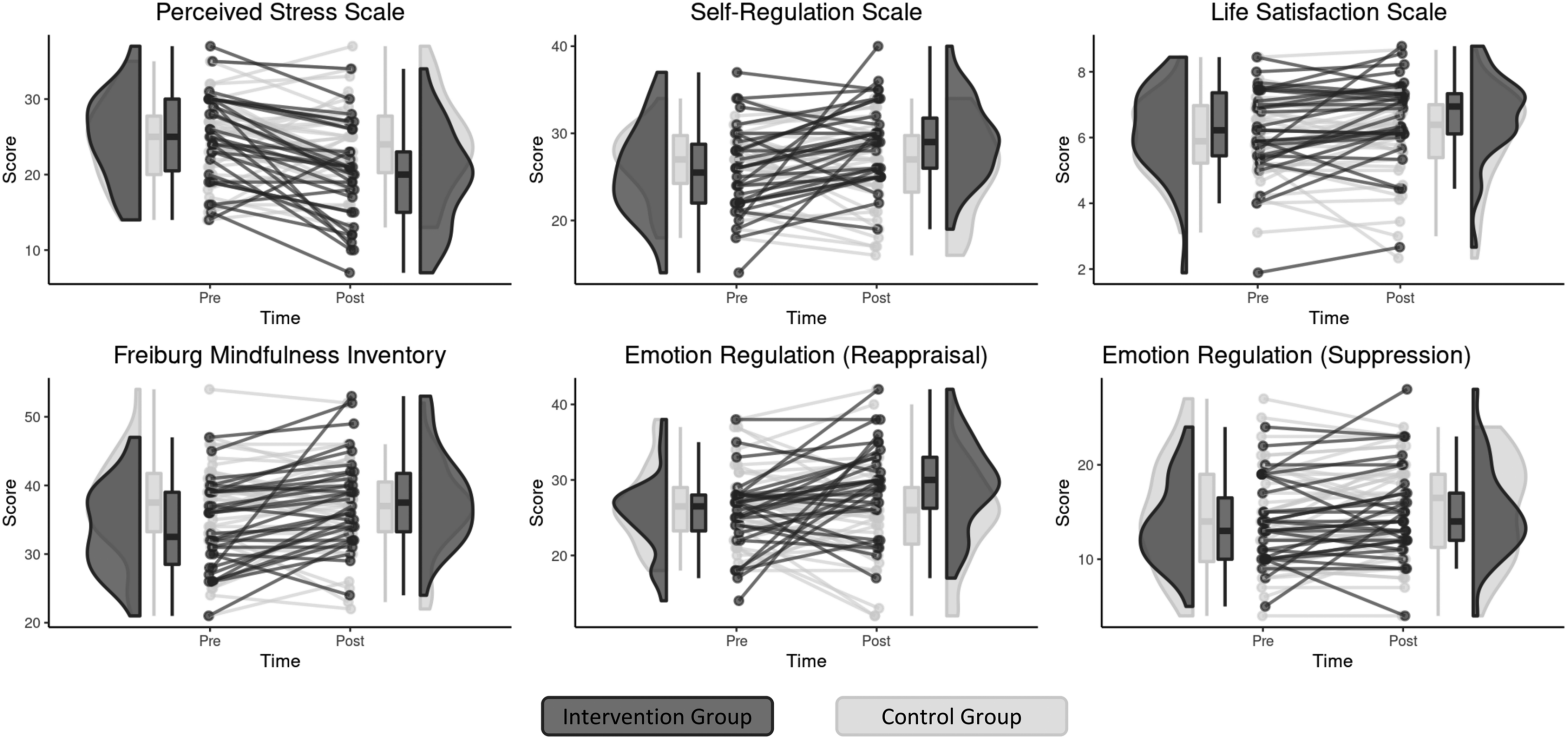
Means of primary and secondary outcomes at baseline and post‐intervention per group

Descriptive results at baseline and post‐intervention per group and time point are depicted in Figure 2. A table specifying all descriptive results is available in the Supporting Information to this article. Overall intervention efficacy on primary outcomes was investigated using a MANOVA with time as the within‐subject factor and group as the between‐subject factor. Outcomes were first evaluated in a multivariate test including all primary outcome variables. To investigate the individual contribution of each variable, univariate effects are reported subsequently.

As predicted, the interaction of group and time was significant in the multivariate model (see Table 2 for details). Figure 2 indicates that these interactions were qualified by relative increases in the intervention group over time, indicating an effect of the mindfulness‐based intervention. Concordantly, univariate tests demonstrated significant improvements in perceived stress and self‐regulation scores, whereas neither the life satisfaction total score nor the two subscales did show significant interactions of time and group.

**TABLE 2:** Multivariate and univariate effects of group and time for primary outcomes

Dependent variable	Pillai's trace	Mean square	*df1*	*df2*	*F*	*p*‐value	η_p_^2^	
Multivariate effects								
Intercept	.996		3	60	4933.715	<.001^*^	.996	
Group	.036		3	60	.752	.526	.036	
Time	.249		3	60	6.616	<.001^*^	.249	
Group × time	.255		3	60	6.852	<.001^*^	.255	^+++^
Perceived Stress								
Intercept		68263.579	1	62	1144.773	<.001^*^	.949	
Group		104.516	1	62	1.753	.190	.027	
Time		271.889	1	62	18.945	<.001^*^	.234	
Group × time		195.951	1	62	13.654	<.001^*^	.180	^+++^
Self‐Regulation								
Intercept		91978.041	1	62	2611.745	<.001^*^	.977	
Group		30.760	1	62	.873	.354	.014	
Time		58.346	1	62	5.974	.017^*^	.088	
Group × time		146.939	1	62	15.045	<.001^*^	.195	^+++^
Life Satisfaction, total								
Intercept		4905.751	1	62	1633.718	<.001^*^	.963	
Group		6.131	1	62	2.042	.158	.032	
Time		2.533	1	62	5.530	.022^*^	.082	
Group × time		1.002	1	62	2.188	.144	.034	
Life Satisfaction, current								
Intercept		4017.322	1	62	1238.085	<.001^*^	.952	
Group		4.447	1	62	1.371	.246	.022	
Time		4.813	1	62	3.129	.082	.048	
Group × time		2.875	1	62	1.869	.176	.029	
Life Satisfaction, past								
Intercept		5383.219	1	62	1611.169	<.001^*^	.963	
Group		7.074	1	62	2.117	.151	.033	
Time		1.666	1	62	3.850	.054	.058	
Group × time		.428	1	62	.989	.324	.016	
Mindfulness								
Intercept		167774.142	1	62	2219.874	<.001^*^	.973	
Group		28.767	1	62	.381	.540	.006	
Time		116.618	1	62	8.647	.005^*^	.122	
Group × time		176.618	1	62	13.096	<.001^*^	.174	^+++^
Emotion‐Regulation, reappraisal								
Intercept		91250.938	1	62	1836.539	<.001^*^	.967	
Group		56.251	1	62	1.132	.291	.018	
Time		72.565	1	62	3.871	.054	.059	
Group × time		182.252	1	62	9.723	.003^*^	.136	^++^
Emotion‐Regulation, suppression								
Intercept		27536.777	1	62	586.272	<.001^*^	.904	
Group		16.777	1	62	.357	.552	.006	
Time		34.294	1	62	5.705	.020^*^	.084	
Group × time		.294	1	62	.049	.826	.001	

*Notes*: Multivariate and univariate outcomes of MANOVA. Multivariate model includes all primary outcome variables: Perceived Stress (PSS‐10), Self‐Regulation (SRS‐10) and Life Satisfaction total (LSS‐total). Additionally reported secondary measures: Life Satisfaction subscale current and subscale past (LSS‐current, LSS‐past), Mindfulness (FMI‐14) and Emotion‐Regulation (ERQ‐reappraisal, ERQ‐suppression).

^*^ *p*‐value < .05.

^+^ ηp2 > .01 (small effect).

^++^ ηp2 > .06 (medium effect).

^+++^ ηp2 > .14 (large effect).

Regarding secondary outcomes, univariate group‐by‐time interactions were also significant for mindfulness scores and the emotion‐regulation subscale reappraisal, but not for the Emotion‐Regulation subscale suppression, which was in line with our expectations. Detailed results for univariate effects can be found in Table 2.

### Mediation and moderation analysis

Mindfulness and emotion regulation were tested as potential mediators of the intervention effect using a within‐subject statistical analysis method (Montoya & Hayes, 2017). Potential mediators were tested for each significant primary outcome variable, hence perceived stress and self‐regulation. As depicted in Table 3, significant indirect mediation effects were found for mindfulness on self‐regulation. Descriptively, the observed intervention effect on reducing perceived stress was larger with increased mindfulness scores; however, the estimated 95% confidence interval was not entirely in the negative range.

**TABLE 3:** Mediation effects of Mindfulness and Emotion‐Regulation on primary outcomes

Primary outcome, mediation path			Bias‐corrected bootstrap 95%CI	
	Effect	BootSE	Lower	Upper
Perceived Stress				
Direct effect	−4.430	1.221	−6.957	−1.903^a^
Mindfulness	−1.574	1.241	−4.102	.823
Emotion‐Regulation, reappraisal	.926	.938	−.319	3.888
Emotion‐Regulation, suppression	−.322	.450	−1.946	.158
Self‐Regulation				
Direct effect	1.260	1.093	−1.000	3.521
Mindfulness	2.727	1.581	.063	6.282^a^
Emotion‐Regulation, reappraisal	−.807	.689	−2.840	.118
Emotion‐Regulation, suppression	.319	.457	−.170	1.896

*Notes*: Mediation effects of Mindfulness (FMI‐14) and Emotion‐Regulation (ERQ‐reappraisal, ERQ‐suppression) on within‐subject differences of Perceived Stress (PSS‐10) and Self‐Regulation (SRS‐10) in the intervention group. Method: Bias‐corrected bootstrap with 5000 samples.

^a^ 95%CI does not include 0 indicating statistical significance.

**FIGURE 3:**
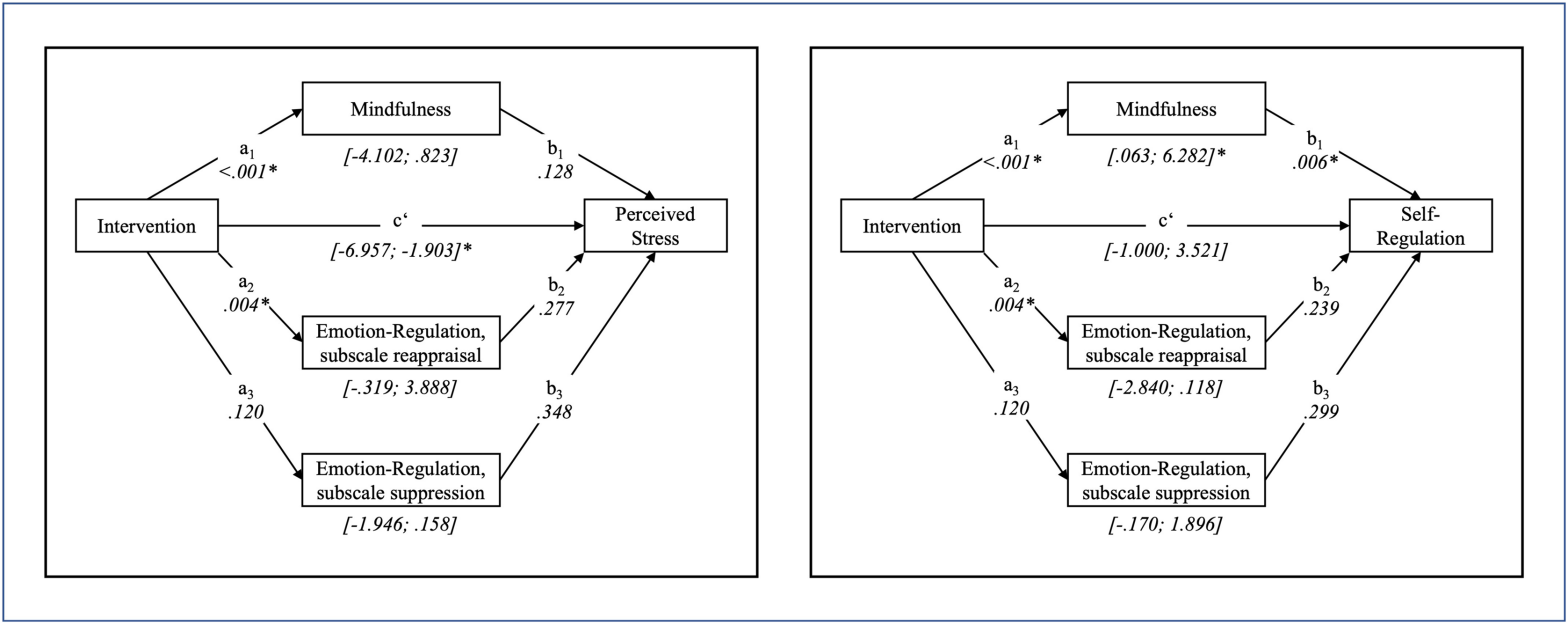
Path analysis of mediative effects of mindfulness on perceived stress and self‐regulation [Color figure can be viewed at wileyonlinelibrary.com]

For self‐regulation as a dependent variable, a significant proportion of the total intervention effect was explained through the indirect path, resulting in non‐significance of the direct effect (see Figure 3).

**FIGURE 4:**
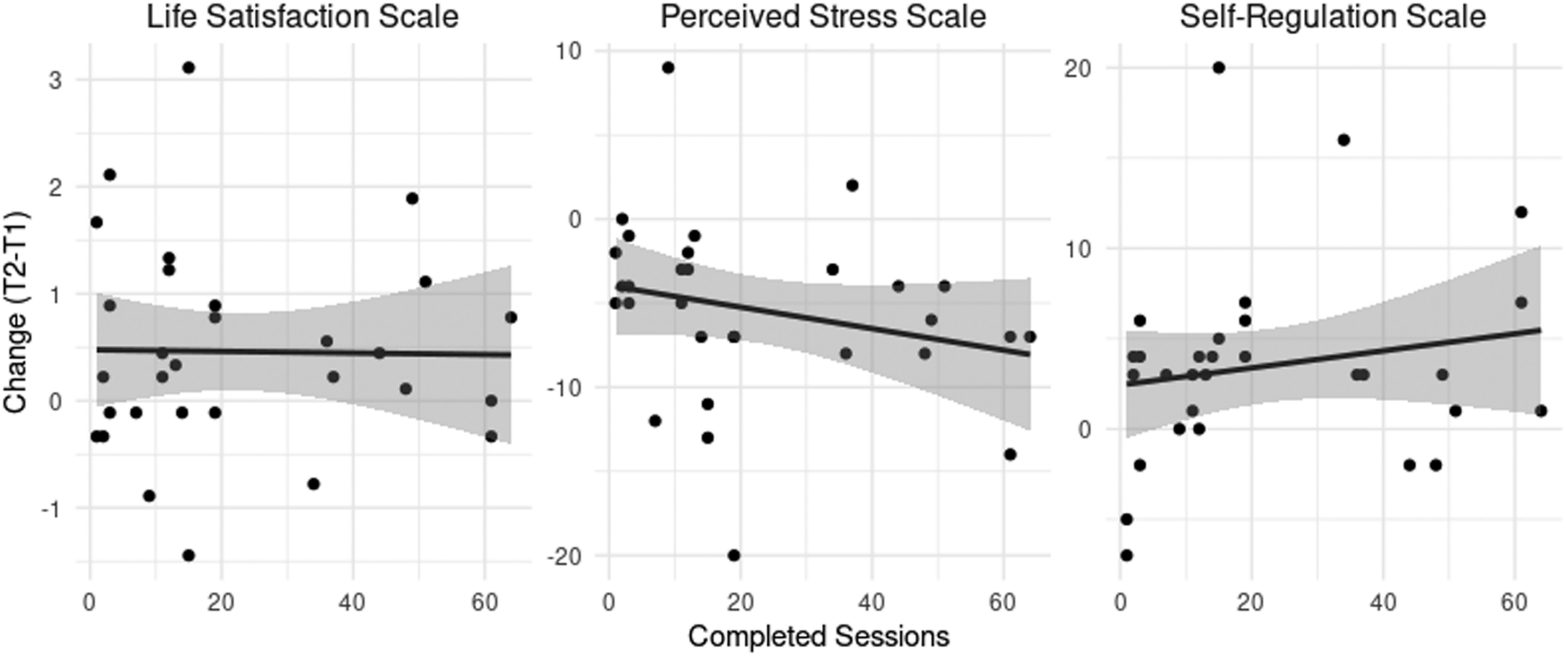
Moderating effects of the amount of completed sessions on primary outcomes

We also tested whether increases in the outcome variables for the intervention group were moderated by the amount of successfully completed intervention sessions per participant (see Figure 4). Engagement in the intervention varied across participants with a mean of 22.53 completed sessions (*SD* = 20.31, *Md* = 14.5, *range*: 1–64). Interaction effects of sessions and each outcome variable over time within the intervention group were not significant. Although effects on perceived stress (*b* = −.064, *t*(28) = −1.329, *p* = .194) and self‐regulation (*b* = .047, *t*(28) = .949, *p* = .351) were descriptively larger with higher amount of sessions, no descriptive difference for life satisfaction (*b* = −.001, *t*(28) = −.083, *p* = .934) was observed.

## DISCUSSION

The purpose of this study was to investigate the effects of a mobile mindfulness‐based intervention on perceived stress, self‐regulation abilities and life satisfaction in a part‐time working student population. Results showed that the intervention was effective in decreasing perceived stress and enhancing self‐regulation, both with large effect sizes. Unexpectedly, however, no significant effects for life satisfaction were found. The study further investigated if a mobile mindfulness‐based intervention has an effect on mindfulness and cognitive reappraisal and if these effects mediate the primary outcomes. Results showed that the intervention was effective in increasing both mindfulness and cognitive reappraisal with medium to large effect sizes. Further, mindfulness was found to fully mediate the intervention effect on self‐regulation, but no reliable mediation was found for perceived stress. Cognitive reappraisal did not significantly mediate either of the primary outcomes. Finally, the study investigated if the amount of completed intervention sessions moderates the primary outcomes. Results showed that the amount of completed sessions did not have a significant effect on changes in the outcome variables. Overall, the app‐based mindfulness intervention seems to be effective in increasing part‐time working students' mental well‐being, attentional and emotional self‐regulation abilities and mindfulness. Although self‐reported mindfulness could partially account for these improvements, no dependency of effects on the amount of completed meditation sessions was found.

Previous studies suggested that improvements in emotion regulation could potentially mediate psychological outcomes of mindfulness‐based interventions (Freudenthaler et al., 2017;Garland et al., 2011; Hanley et al., 2015; Hanley & Garland, 2014). Other than expected, increases in cognitive reappraisal did not explain improvements in perceived stress and self‐regulation outcomes in this study. This raises the possibility that changes in cognitive reappraisal induced by mindfulness‐based training might not directly contribute to improvements in perceived stress and self‐regulations. Alternatively, this deviation could be explained by differences in conceptualisation of reappraisal and investigated outcome variables compared with previous research. In the present study, reappraisal (vs. suppression) was conceptualised as a distinctive mode of emotional processing in which people habitually tend to engage early on in the emotion‐generative process (Gross & John, 2003). Furthermore, we investigated outcome variables of well‐being and cognitive functioning. In contrast, the mentioned previous studies mostly investigated ‘positive’ reappraisal (Garland et al., 2011; Hanley et al., 2015; Hanley & Garland, 2014), a concept focusing on the retrospective reappraisal of major adverse life events, such as a cancer diagnosis or suffering from clinical conditions (Garland et al., 2009; Garnefski et al., 2001). Other studies primarily investigated the mediative role of reappraisal on clinical outcomes such as symptoms of anxiety and depression (Desrosiers et al., 2013; Freudenthaler et al., 2017). Thus, it is conceivable that changes in cognitive reappraisal might not contribute directly to improved well‐being, but indirectly through specific reappraisals of past negative life events, or in participants with more severe mental suffering such as depression and anxiety. In line with these interpretations, mindfulness and cognitive reappraisal do not map directly onto each other conceptually, but mindfulness is rather assumed to support positive reappraisal in a specific way by enhancing awareness of and decentring from thoughts, broadening attention, and thereby allowing to appraise stressful events as benign, beneficial, or meaningful (Garland et al., 2011).

In line with our expectations and current theories on active mechanisms of mindfulness‐based interventions (Lindsay & Creswell, 2017), increases in mindfulness qualified increases in self‐regulation. However, no such significant mediation was found for perceived stress. Because previous studies strongly advocate for the mediative role of mindfulness in the effect of mindfulness‐based interventions on perceived stress (Desrosiers et al., 2014; Gu et al., 2015; Hoffmann & Geisler, 2020; Lindsay & Creswell, 2017), these findings might indicate that the sample size was too small to establish significance for this particular mediation link with the chosen test design. This is also indicated by the fact that, however non‐significant, the confidence interval of the observed effect was mostly in a negative range, descriptively supporting the mediation of reductions in perceived stress through mindfulness.

Sample size might also play a role in the non‐significance of intervention effects on life satisfaction. Again, small descriptive improvements in life satisfaction and especially current life satisfaction could be observed, indicating that sample power was not strong enough to establish significance for this particular outcome with the chosen test design. Other studies on app‐based mindfulness programmes with similar sample sizes have found mixed results, reporting no significant (Howells et al., 2016) or significant results with large confidence intervals (Champion et al., 2018). Recent meta‐analyses on conventional mindfulness‐based intervention programmes have also reported mixed results. One study reported no significant results for life satisfaction in university students (Dawson et al., 2020); two others found small to medium effects for life satisfaction when reported together with other variables as ‘(positive) well‐being’ in working adult populations (Lomas et al., 2019; Vonderlin et al., 2020). As changing life satisfaction is a process including not only increased attention to affects but also an evaluative judgemental component (Pavot & Diener, 2008), it is possible that specific course components of mindfulness‐based interventions might be especially effective in increasing life satisfaction and others focusing on acceptance might interfere with re‐evaluating life satisfaction (Lindsay & Creswell, 2017). Further, because judgements of life satisfaction are highly influenced by evaluation of and successes in rather stable life domains such as academic performance, romantic relationships and family relationships (Diener & Seligman, 2002; Schimmack & Oishi, 2005), a wider time frame of observation could unravel significant changes in participants life satisfaction. A follow‐up study could employ a time‐series analysis investigating developments in outcomes over time during and after the intervention. Such a design might also help to disentangle the effects of the various course components (i.e. practices focussed on presence, body‐awareness and loving‐kindness) on the various outcomes (perceived stress, self‐regulation, life satisfaction, etc.).

Finally, no significant dose–response effect of the amount of completed sessions on primary outcome variables was found. Surprisingly, this is in line with findings from recent meta‐analyses reporting no significant effect of practice frequency on psychological outcomes in mindfulness interventions (Blanck et al., 2018; Schumer et al., 2018; Strohmaier, 2020). A possible explanation is that effects of mindfulness‐based interventions might not accumulate linearly, but rather as a function of the participants' informal application of learned mindfulness skills in their daily life. This might also explain why some participants already benefited from completing only a few formal sessions, whereas others who completed many sessions did not benefit significantly more. Another important factor influencing outcome effects might be practice quality rather than frequency (Goldberg et al., 2020). Participants who are encouraged to engage deliberately and with effort in their meditation practice, who are motivated and committed to discover themselves and understand the theoretical foundations of their activity, might benefit more from practice time than others. However, these explanations remain speculative. Given that in our trial descriptive tendencies for a dose–response relationship were observed, a strongly powered study might contrarily find significance. Future research additionally tracking informal practice, daily application of mindfulness skills and practice quality through advanced methods (e.g. ecological momentary assessment) might clarify this relationship.

Several limitations apply to the design of this study. First, as discussed earlier, the sample size may have been too small to detect weaker effects of the intervention on life satisfaction and the mediative effect of mindfulness on perceived stress. Considering this study was a first exploration of efficacy in a newly specified population with an app‐based intervention that has never been tested before, the sample size was within the standards of the field (Goldberg et al., 2017). Second, attrition due to non‐responsiveness (*n* = 24) throughout the intervention phase was a strong contributor to the final sample size, raising the question of self‐selection that would limit generalisability of the study's findings. However, participants in the dropout group did not differ from the analysed sample in demographic variables and baseline outcomes. High dropout rate can presumably be attributed to the rather impersonal online setting of the study and the non‐provision of incentives for continued participation (O'Neil et al., 2003) as well as the fact that this is an extremely busy sample. Finally, the presented study employed a wait‐list controlled rather than an actively controlled design. Future studies could implement additional control groups that receive treatments not containing active mindfulness practice.

The overall positive findings of the presented study for the efficacy of a mobile mindfulness‐based programme compared with a wait‐listed control group raise the question if the applications efficacy is also comparable to traditional forms of delivery, as this would open up new opportunities to low‐cost digital healthcare interventions for pre‐clinical patients in early stages of their symptom trajectories. Future research targeting this question could employ multiple non‐clinical, pre‐clinical and clinical samples that either receive conventional forms of mindfulness‐based programmes or mobile interventions that were found to be effective in previous studies. Future studies could also tackle the question whether there are specific parts of the intervention that are especially beneficial to specific groups or that only develop an effect over time. To answer this, studies could involve groups that only receive specific parts of the mobile intervention to assess dose–response relationships and effect mechanisms and observe these over a longer period of time to detect effects that unravel only in the long term.

In summary, this randomised controlled trial demonstrated that an app‐based mindfulness intervention is effective in improving mental well‐being and coping ability in part‐time working students. Specifically, the study found the intervention to effectively reduce perceived stress and increase self‐regulation, mindfulness and cognitive reappraisal. Further, the study showed that beneficial outcomes could partly be explained by increased mindfulness. Future studies should continue investigating the pathways and active components of the treatment. This may allow tailoring the intervention to serve the needs of specific populations. Evidence‐based complexity models on how mindfulness‐based interventions influence outcomes under given conditions could help to increase the efficacy of brief self‐administered programmes and their applicability in non‐clinical contexts.

## CONFLICT OF INTEREST

We have no conflict of interest to disclose.

## ETHICAL STATEMENT

This trial was performed in accordance with the ethical standards of the German Association of Psychologists. All criteria for low‐risk classification as defined by the FOM University of Applied Sciences ethical guidelines were met, thus further committee approval was not required. Participants were fully informed about the aims and procedures of this trial and informed consent was obtained a priori.

## Supporting information


**Table S1.** Mindfulness‐based intervention plan delivered through the Balloon application
**Table S2**. Descriptive results per group at baseline and post‐intervention

## Data Availability

Data supporting the findings of this study are openly available in the Open Science Framework repository at https://doi.org/10.17605/OSF.IO/WUJSG.
