# Supplementary material for: App‐based mindfulness meditation reduces perceived stress and improves self‐regulation in working university students: A randomised controlled trial
Source: Appl Psychol Health Well Being. 2021 Dec 27;14(4):1151–71. doi: 10.1111/aphw.12328 (PMC9788174; doi:10.1111/aphw.12328)
Supplement: Supplementary file 1 — Table S1. Mindfulness‐based intervention plan delivered through the Balloon application Table S2. Descriptive results per group at baseline and post‐intervention [file APHW-14-1151-s001.docx]

**Supplements I**

*Mindfulness-based intervention plan delivered through the Balloon application*

| Translated title  *(original title)* | Description | Number of sessions | Total duration, in minutes |
| --- | --- | --- | --- |
| Introduction *(Einstiegskurs)* | A brief 7-day introductory course elaborating major principles of mindfulness, mindful meditation, and giving an outlook on how mindful activities can be of use in everyday life. | 7 | 57 |
| Mindful Living I *(Achtsam leben I)* | First part of a two-tiered basic course discussing fundamental principles of living mindfully and methods of practicing mindfulness, such as breath-focused meditation or meta-awareness. | 10 | 115 |
| Reducing Stress *(Stress reduzieren)* | A course elaborating basic theoretical foundations of stress, daily hassles and overstraining, and providing mindfulness-based methods of coping. | 10 | 119 |
| Being Happy *(Glücklich sein)* | Mindfulness-based exercises and educational discussions about happiness in life as the result of a benevolent attitude towards life and a positive relationship to oneself and others. | 12 | 137 |
| Understanding Thoughts *(Gedanken verstehen)* | A course focussing on meta-awareness and meta-cognition, presenting methods of recognizing and interpreting thoughts from a observatory position (cognitive defusion). | 8 | 91 |
| Self-Compassion *(Für sich sorgen)* | Elaboration of self-compassion and self-awareness as basis for a positive attitude towards life and a benevolent relation to oneself. Methods of introspection, reflection and self-care are being taught. | 10 | 119 |
| Total |  | 57 | 638 |

**Supplements II**

*Descriptive results per group at baseline and post-intervention*

| Dependent Variable | Baseline | | | | |  | Post-Intervention | | | | |  |
| --- | --- | --- | --- | --- | --- | --- | --- | --- | --- | --- | --- | --- |
|  | Intervention Group (n = 30) | |  | Control  Group  (n = 34) | |  | Intervention Group  (n = 30) | |  | Control  Group (n = 34) | |  |
|  | *M* | *SD* |  | *M* | *SD* |  | *M* | *SD* |  | *M* | *SD* |  |
| *Primary outcome variables* |  |  |  |  |  |  |  |  |  |  |  |  |
| Perceived Stress | 24.93 | 5.88 |  | 24.26 | 5.73 |  | 19.53 | 6.55 |  | 23.82 | 6.17 |  |
| Self-Regulation | 25.60 | 5.26 |  | 26.76 | 3.85 |  | 29.10 | 4.63 |  | 25.97 | 5.16 |  |
| Life Satisfaction, total | 6.19 | 1.37 |  | 5.93 | 1.18 |  | 6.65 | 1.29 |  | 6.04 | 1.41 |  |
| Life Satisfaction, subscale current | 5.46 | 1.53 |  | 5.38 | 1.44 |  | 6.14 | 1.49 |  | 5.47 | 1.71 |  |
| Life Satisfaction, subscale past | 6.56 | 1.41 |  | 6.21 | 1.29 |  | 6.91 | 1.33 |  | 6.32 | 1.45 |  |
| *Secondary outcome variables* |  |  |  |  |  |  |  |  |  |  |  |  |
| Mindfulness | 33.67 | 6.29 |  | 36.97 | 6.98 |  | 37.93 | 6.73 |  | 36.53 | 6.64 |  |
| Emotion-Regulation, subscale reappraisal | 25.47 | 5.16 |  | 26.53 | 5.28 |  | 29.37 | 5.88 |  | 25.65 | 6.85 |  |
| Emotion-Regulation, subscale suppression | 13.77 | 4.45 |  | 14.59 | 5.64 |  | 14.90 | 4.92 |  | 15.53 | 5.40 |  |
| *Note*. Descriptive statistics per group, reported as Mean (M) and standard deviation (SD) at baseline and post-intervention. Included measures: Perceived Stress (PSS-14), Self-Regulation (SRS-10), Life Satisfaction (LSS-total, LSS-current, LSS-past), Mindfulness (FMI-14) and Emotion-Regulation (ERQ-reappraisal, ERQ-suppression). | | | | | | | | | | | |  |
